# Genome-wide investigation of UDP-Glycosyltransferase family in Tartary buckwheat (*Fagopyrum tataricum*)

**DOI:** 10.1186/s12870-024-04926-8

**Published:** 2024-04-06

**Authors:** Fan Yang, Lei Zhang, Xiao Zhang, Jingru Guan, Bo Wang, Xiaoying Wu, Minli Song, Aili Wei, Zhang Liu, Dongao Huo

**Affiliations:** 1 College of Biological Sciences and Technology, Taiyuan Normal University, Taiyuan, 030619 China; 2 MARA Key Laboratory of Crop Ecophysiology and Farming System in the Middle Reaches of the Yangtze River, College of Plant Science and Technology, Huazhong Agricultural University, Wuhan, 430070 China; 3 Center for Agricultural Genetic Resources Research, Shanxi Agricultural University, Taiyuan, 030031 China

**Keywords:** Fagopyrum tataricum, UGT, Genome-wide analysis, Expression pattern

## Abstract

**Background:**

Tartary buckwheat (*Fagopyrum tataricum*) belongs to Polygonaceae family and has attracted increasing attention owing to its high nutritional value. UDP-glycosyltransferases (UGTs) glycosylate a variety of plant secondary metabolites to control many metabolic processes during plant growth and development. However, there have been no systematic reports of UGT superfamily in *F. tataricum*.

**Results:**

We identified 173 FtUGTs in *F. tataricum* based on their conserved UDPGT domain. Phylogenetic analysis of FtUGTs with 73 *Arabidopsis* UGTs clustered them into 21 families. FtUGTs from the same family usually had similar gene structure and motif compositions. Most of *FtUGTs* did not contain introns or had only one intron. Tandem repeats contributed more to *FtUGTs* amplification than segmental duplications. Expression analysis indicates that *FtUGTs* are widely expressed in various tissues and likely play important roles in plant growth and development. The gene expression analysis response to different abiotic stresses showed that some *FtUGTs* were involved in response to drought and cadmium stress. Our study provides useful information on the *UGTs* in *F. tataricum*, and will facilitate their further study to better understand their function.

**Conclusions:**

Our results provide a theoretical basis for further exploration of the functional characteristics of *FtUGTs* and for understanding the growth, development, and metabolic model in *F. tataricum.*

**Supplementary Information:**

The online version contains supplementary material available at 10.1186/s12870-024-04926-8.

## Introduction

Glycosylation refers to a post-translational modification process that alters the chemical property, subcellular location, and biological activity of various biological molecules [1]. It plays a crucial role in the biosynthesis of plant natural secondary metabolites, and also serves as a fundamental mechanism for maintaining intracellular stability. Glycosyltransferases (GTs) constitute a diverse and complex multigene family that facilitates glycosylation by catalyzing the transfer of activated sugar molecules from donor molecules to specific receptor molecules [2–5]. The GTs are divided into 115 families (CAZy, http://www.cazy.org), with GT family 1 (GT1) being the most abundant and widely distributed in plants [6].

GT1, also known as UDP glycosyltransferases (UGTs), facilitate the transfer of the glycosyl group from UDP sugar to various receptors, such as flavonoids, terpenoids, auxin, cytokinins, and salicylic acid [7, 8]. UGTs are ubiquitously present in diverse life forms, ranging from plants and animals to fungi, bacteria, and viruses [9]. UGTs typically utilize UGT sugars such as UDP rhamnose, UDP arabinose, UDP glucose, UDP xylose, and UDP galactose as donor molecules during the glycosylation process [10–13]. The overall plant UGTs included the conserved domain UDPGT domain (PF00201). The majority of plant UGTs share recognition specificity for the same or similar UGT sugar donors at their C-terminal region. This region contains a highly conserved motif referred to as plant secondary product glycosyltransferase (PSPG), which is composed of 44 amino acids (WAPQ–VL-H-AVG-FLTHCGWNSTLES—GVP—WPM–DQ) [14, 15]. The N-terminal region of UGTs exhibits high variability and is capable of recognizing to specific receptors [16]. As a result, the C-terminal region of UGTs is generally considered more conserved than the N-terminal region [17]. .

The *UGT* family has been identified in several plant species [10, 18–26]. The glycosylation process mediated by UGTs is a critical mechanism that maintains the diversity of metabolites in plants. It performs essential functions related to numerous aspects of plant growth and development, including seed germination, growth, flowering, seed setting, and stress resistance [27–37]. *Bronze1*, the initial gene identified in *UGT* superfamily of plants, is capable of synthesizing flavonoid glycosides and regulating the melanin accumulation in maize grains [38, 39]. In grape, *VvGT7* is involved in the biosynthesis of geranyl and neryl glucoside during fruit development [34]. In strawberry, *FaGT2* and *FaGT5* promote the production of β-glucogallin [35]. In wheat, *TaUGT3*and *TaUGT12887* are closely related to scab resistance [28, 32]. *UGT85K4 and UGT85K5* are also involved in the biosynthesis of linolenin and thymosides in cassava, which may contribute to defense response [30]. Overexpression of *AtUGT85A5* enhances plant tolerance to salt stress [33]. *AtUGT73b3* and *AtUGT73b5* show resistance to *Pseudomonas syringae* [27]. Knockout of *Arabidopsis UGT76B1* can lead to increased resistance to *Pseudomonas syringae* and accelerated senescence [31].

*UGT* family are named following the conventions of the UGT Nomenclature Committee. This involves the use of the root symbol UGT, followed by a numerical value representing a specific family. The range of families varies among different organisms. In animals, the family names range from 1 to 50, in yeast it ranges from 51 to 70, in plants it ranges from 71 to 100, and in bacteria the range is from 101 to 200 [40, 41].

Tartary buckwheat (*Fagopyrum tataricum*) belongs to the Polygonaceae family and is a gluten-free crop that provides not only energy but also a variety of beneficial bioactive compounds [42]. Previous research has indicated that Tartary buckwheat possesses a significant quantity of antioxidants, especially flavonoids and polyphenols [43, 44]. These bioactive compounds have been linked to numerous health benefits, including cholesterol reduction, neuroprotection, anti-inflammatory, anti-diabetic, anti-hypertensive, and anti-cancer effects [45–47]. As a result, Tartary buckwheat has been recognized as a functional food [48]. The glycosylation process plays a crucial role in the biosynthesis of these bioactive components [49, 50].

To date, there have been no comprehensive explorations of the UGT superfamily in *F*. *tataricum*. Consequently, this study aimed to undertake a genome-wide investigation into the evolutionary characteristics and biological functions of *F. tataricum UGTs* (*FtUGTs*). To this end, we performed whole genome identification, phylogenetic relationships, conserved motifs, gene structure, cis-acting elements, gene duplication, gene expression, and response to different abiotic stresses of *FtUGTs*. This study will provide a theoretical basis for the further in-depth mining of *UGT* functions in *F. tataricum*.

## Results

### Identification of UGT genes in F. Tataricum

In the present study, The conserved UDPGT domain and 44 aa length PSPG motif was used to identify the presence of *UGTs* in the *F. tataricum* genome. Ultimately, a total of 173 *FtUGT* genes were identified and labeled as *FtUGT1* to *FtUGT173* based on their position in chromosomes (Additional File 1: Table S1). The fundamental characteristics of the 173 FtUGT proteins, such as protein length, protein molecular mass, isoelectric point (pI), domain information and predicted subcellular localization, were thoroughly investigated (Additional file 1: Table S1). The smallest proteins were FtUGT62 and FtUGT87, which consisted of 123 amino acids. Meanwhile, the largest protein was FtUGT35 containing 645 amino acids. The majority of FtUGT proteins had a size range of 400 to 500 amino acids, with only a few exceeding 500 amino acids. The molecular weights of the FtUGTs ranged from 13.52 kDa (FtUGT62) to 71.05 kDa (FtUGT35), while their isoelectric points varied from 4.46 (FtUGT62) to 9.36 (FtUGT162). Based on the results from the predicted subcellular localization analysis, out of the 173 identified FtUGTs, 81 (46.8%) were predicted to be located in the chloroplast region, 64 (37%) in the cytoplasm, 18 (10.4%) in the nucleus, three (1.7%) in the extracellular region, three (1.7%) in the vacuole, two (1.2%) in the mitochondria, one (0.6%) in the cytoskeleton, and one (0.6%) in the peroxisome (Additional File 1: Table S1). The subcellular localization pattern of FtUGTs is consistent with the glycosylation function.

### Phylogenetic analysis of FtUGTs

**Fig. 1:**
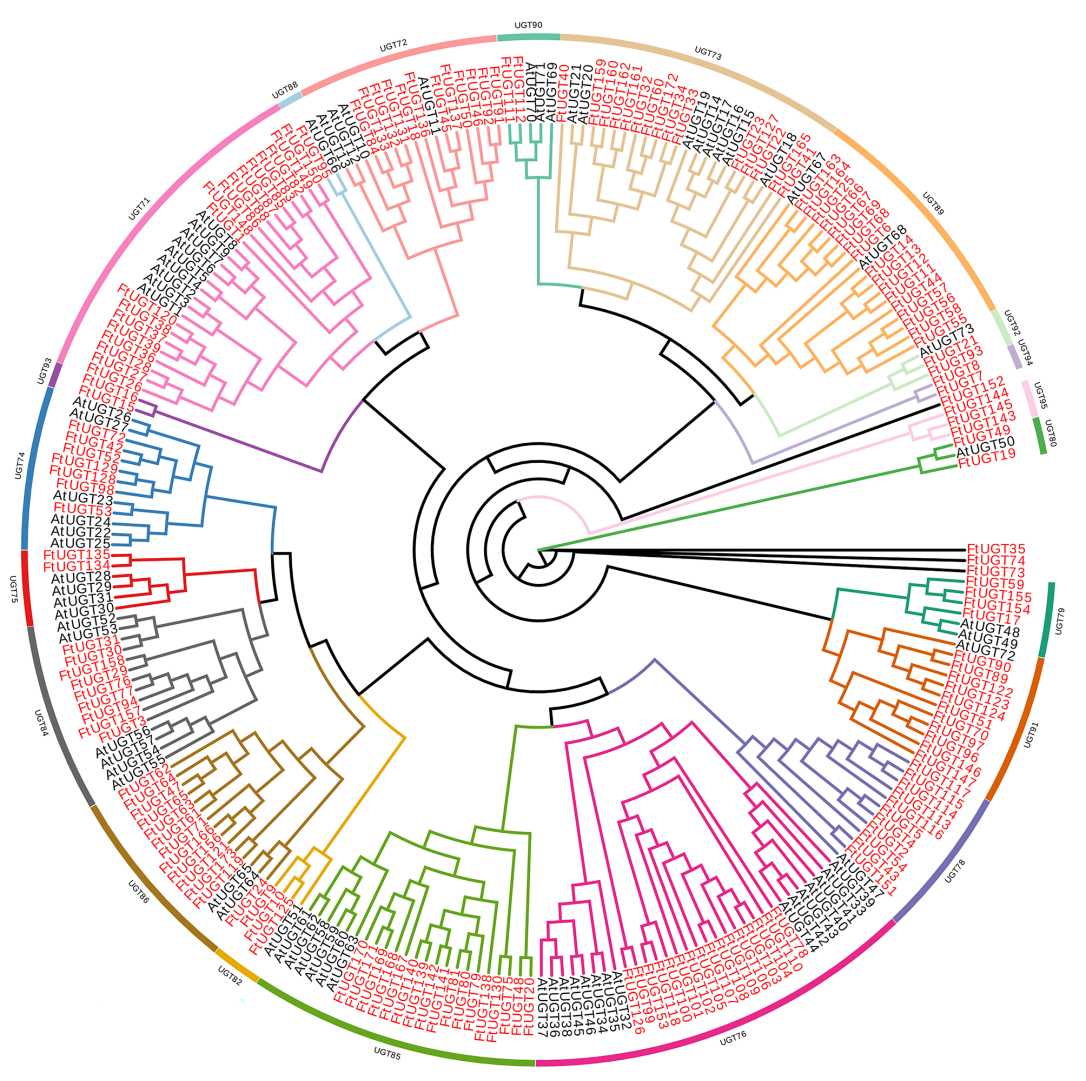
Unrooted phylogenetic tree representing relationships among UGTs of F. tataricum and A. thaliana. The tree was generated using the NJ method in MEGA11.0, and UGT proteins from A. thaliana were prefixed with ‘At’

To analyze the evolutionary relationships between 173 identified FtUGTs and 73 AtUGTs, a phylogenetic tree was constructed using the neighbor-joining (NJ) method with a bootstrap value of 1000, based on their amino acid sequences. (Fig. 1, Additional file 1: Table S1). Based on the classification method proposed by Mackenzie and Yonekura-Sakakibara [39, 40], 246 UGT proteins in the phylogenetic tree were divided into 21 branches (families) and each family was greater than 40% sequence identity. Among the 21 UGT families, 15 UGT families were shared by *A. thaliana*, 6 families (UGT80, UGT82, UGT92, UGT93, UGT94 and UGT95) were additional and 2 families (UGT83 and UGT87) were lacking. The number of FtUGTs in each family varied, as UGT71, the largest of the families, contained 18 UGT members, while UGT88, the smallest of the families, had only one member. There were 17, 17, 16, 15, 13, 12, 11, 11, 9, 7, 4, 3, 3, 2, 2, 2, 2, 2, and 2 FtUGTs in the UGT85, UGT89, UGT76, UGT73, UGT86, UGT72, UGT78, UGT91, UGT84, UGT74, UGT79, UGT82, UGT95, UGT75, UGT80, UGT90, UGT92, UGT93, and UGT94, respectively (Fig. 1, Additional file 1: Table S1). The phylogenetic tree including both *F. tataricum* and *A. thaliana* UGTs revealed that FtUGTs clustered closely with AtUGTs. This supports the notion that these proteins likely share similar physiological functions.

### Gene structure, conserved motifs and cis-elements analysis of FtUGTs

**Fig. 2:**
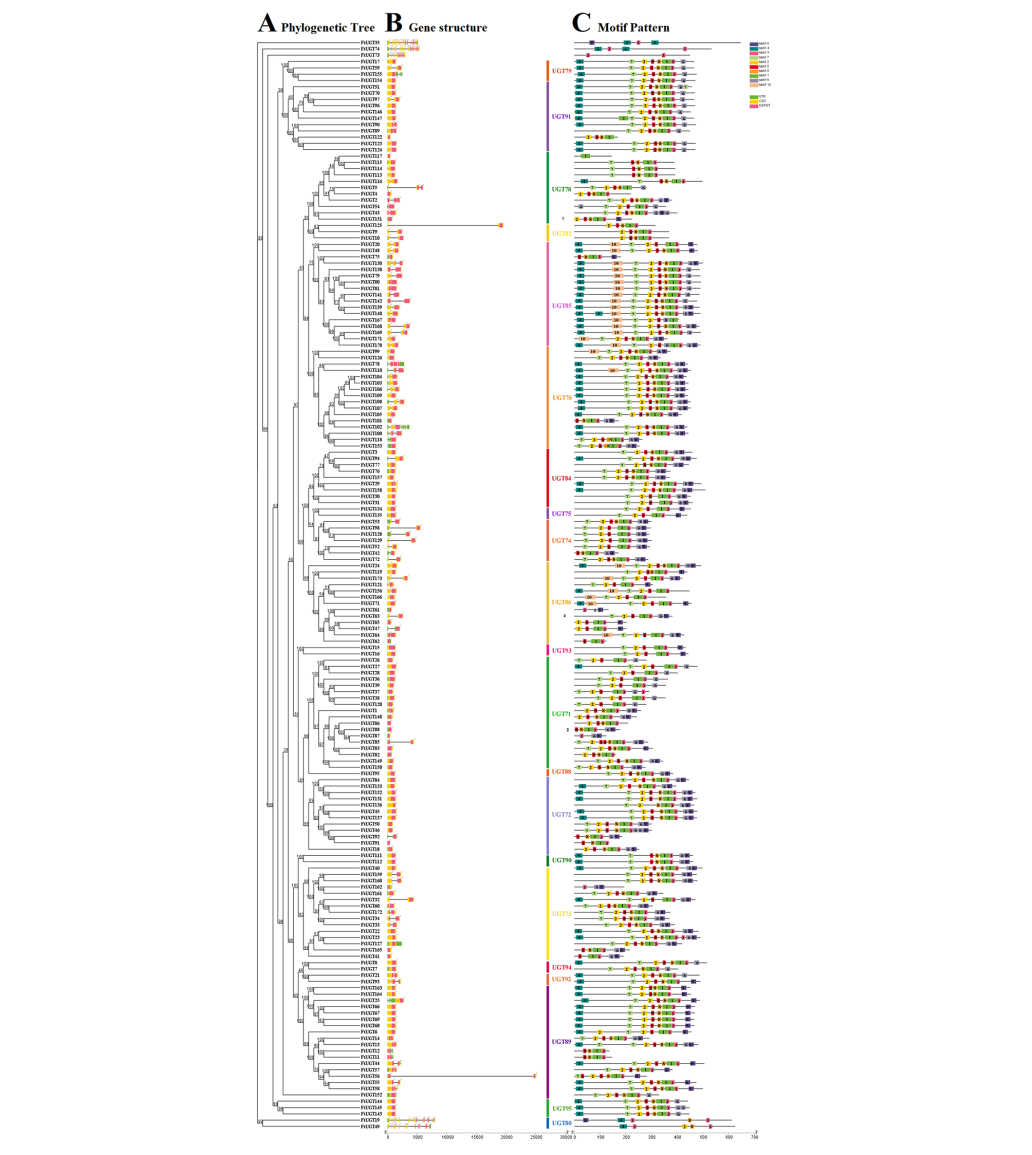
Conserved motifs and gene structure analysis of *UGTs* in *F. tataricum.***A**. Phylogenetic tree was constructed by the NJ method. **B**. Gene structure of *FtUGTs*. Exons and introns are indicated by yellow rectangles and gray lines, respectively. **C**. Amino acid motifs in the FtUGT proteins are represented by colored boxes

As multigene families evolve, newly generated copies often acquire novel gene functions, which can be observed through the diversification of gene structures in the FtUGT superfamily. To gain a more comprehensive understanding of the structural divergence among various *FtUGT* genes, their exon and intron structures were examined by analyzing their genome locations. (Fig. 2, Additional files 1: Tables S1). We found that they had different numbers of introns, ranging from 0 to 13 (Fig. 2). 70 *FtUGT* genes (40.5%) did not contain introns; 72 *FtUGT* genes (41.6%) contained one intron; 23 *FtUGT* genes (13.3%) contained two introns; and 8 *FtUGT* genes (4.6%) contained more than two introns. These results indicated that most members of the *FtUGT* superfamily had simple gene structures and less than three introns, which may be one of the reasons for the high activity and rapid transcription of the UGT superfamily. Generally, *FtUGTs* belonging to the same family exhibited similar gene structures and intron compositions. For example, UGT72, UGT73, UGT74, UGT75, UGT82, UGT84, UGT88, UGT90 UGT91, UGT93, UGT94, and UGT95 family members contained 0 or 1 intron, and UGT80 contained 13 introns. As an illustration, members of UGT72, UGT73, UGT74, UGT75, UGT82, UGT84, UGT88, UGT90, UGT91, UGT93, UGT94, and UGT95 families exhibited either zero or one intron, while UGT80 family members contained 13 introns.

An analysis of the characteristic amino acid regions of FtUGTs involved the investigation of their motifs through the use of the MEME online software. This resulted in the identification of ten conserved motifs, labeled as motifs 1–10. (Fig. 2C, Additional file 2: Table S2). As shown in Fig. 2C, motif 3 was commonly found in FtUGTs, except for FtUGT117 and FtUGT122, and was typically located in close proximity to motif 1. Certain motifs were observed to occur exclusively in specific positions. For example, Motifs 4 and 7 were consistently found at the N-terminus of FtUGTs, while motif 9 was almost always located at the C-terminus. Members of the same FtUGT family typically exhibited similar motif compositions. For instance, UGT75 contained motifs 7, 2, 5, 1, 3, and 9; UGT82 contained motifs 2, 5, 8, 1, and 3; and UGT79, UGT92, and UGT95 all shared motifs 4, 7, 2, 5, 8, 1, 3, and 6.

As promoter cis-elements are closely associated with gene function, this study aimed to investigate the regulatory mechanisms of *FtUGTs* by analyzing the cis-elements within their promoter regions (up to 2000 bp). Using this approach, a total of 120 cis-regulatory elements were identified in 173 *FtUGTs* and were divided into eight categories—light-responsive, development-related, site binding-related, hormone-responsive, promoter-related, environmental stress-related, and other elements (Additional file 3: Table S3). Promoter-related elements CAAT-box and TATA-box, light-response elements TCT motif, GT1 motif, Box 4, G-box and I-box and drought-responsive elements MYB and MYC were identified in nearly all *FtUGTs*. Moreover, a total of 13 hormone-responsive cis-elements were detected amongst *FtUGTs*, encompassing a broad range of plant hormones, including auxin-responsive elements (AuxRR-core, TGA-element, AuxRE, TGA-box), gibberellin-responsive elements (P-box, GARE-motif, P-box, TATC-box), abscisic acid-responsive elements (ABRE), jasmonic acid-responsive elements (TGACG-motif, CGTCA-motif), and salicylic acid-responsive elements (TCA-element, SARE). Almost 96% of the *FtUGTs* contained hormone-responsive elements. Specifically, 80 *FtUGTs* contained IAA-responsive elements, 94 contained GA-responsive elements, 141 contained ABA-responsive elements, 140 contained JA-responsive elements, and 68 contained SA-responsive elements. Significantly, 16 *FtUGTs* possessed all five hormone-responsive elements. In addition, the identified cis-elements within the promoter regions of *FtUGTs* included those associated with environmental stress response, such as drought, salt, low-temperature, anaerobic, and wound stress. These observations collectively indicate that *FtUGTs* participate in the regulation of diverse biological processes in *F. tataricum*, such as plant growth and development, light response, hormone signaling, and abiotic stress adaptation.

### Distribution and duplication of FtUGTs

**Fig. 3:**
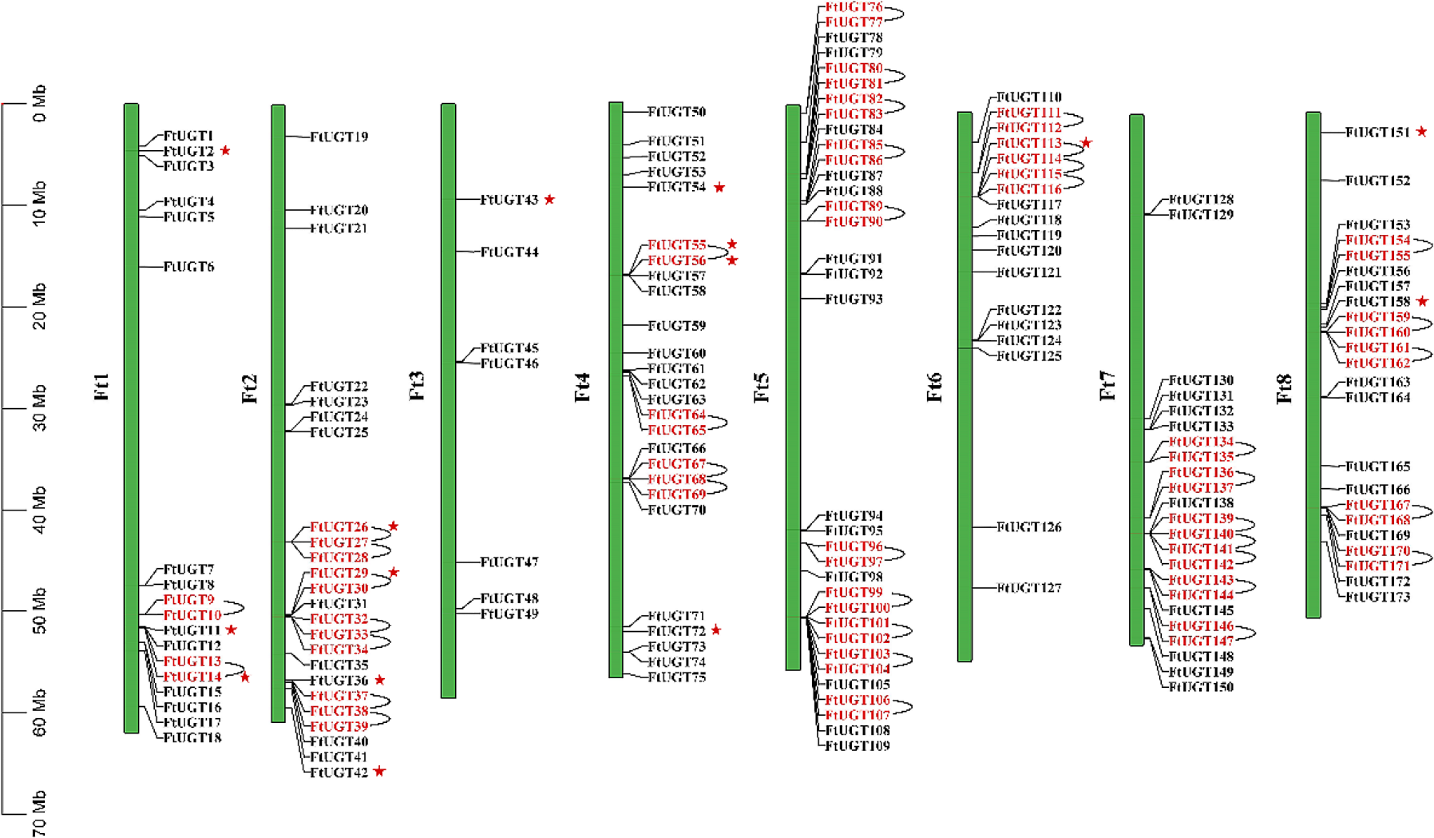
Chromosome mapping and duplication of *FtUGTs* in *F. tataricum*. The chromosome number is indicated on the left side of the chromosome and on the right is the gene ID. Tandem duplication gene pairs are marked with red font and segmental duplication gene pairs are marked with red stars

Utilizing the *F. tataricum* genome database, a physical map was generated to display the positions of the *FtUGTs* across the genome (Fig. 3, Additional files S1 and S4: Tables S1 and S4). Overall, the 173 *FtUGTs* were observed to be non-uniformly dispersed throughout the eight chromosomes of *F. tataricum*. Amongst the chromosomes, Chr5 had the highest gene count (34 genes), followed by Chr4 (26 genes), whereas Chr3 had the lowest gene count (7 genes). Additionally, the analysis revealed a total of 39 tandem duplication events involving 70 FtUGTs (Fig. 3). Intriguingly, eight genes were found to be associated with two tandem repeat events (*FtUGT27* and *FtUGT26*/*FtUGT28*, *FtUGT33* and *FtUGT32*/*FtUGT34*, *FtUGT38* and *FtUGT37*/*FtUGT39*, etc.). The presence of these duplications suggested that certain chromosomal regions might have concentrated tandem duplication events. Moreover, it was observed that tandem duplication pairs were usually members of the same UGT family. Notably, the UGT71 and UGT89 families in particular exhibited a higher frequency of tandem duplication events, indicating that they may have played a significant role in driving the expansion of the UGT superfamily in *F. tataricum*.

**Fig. 4:**
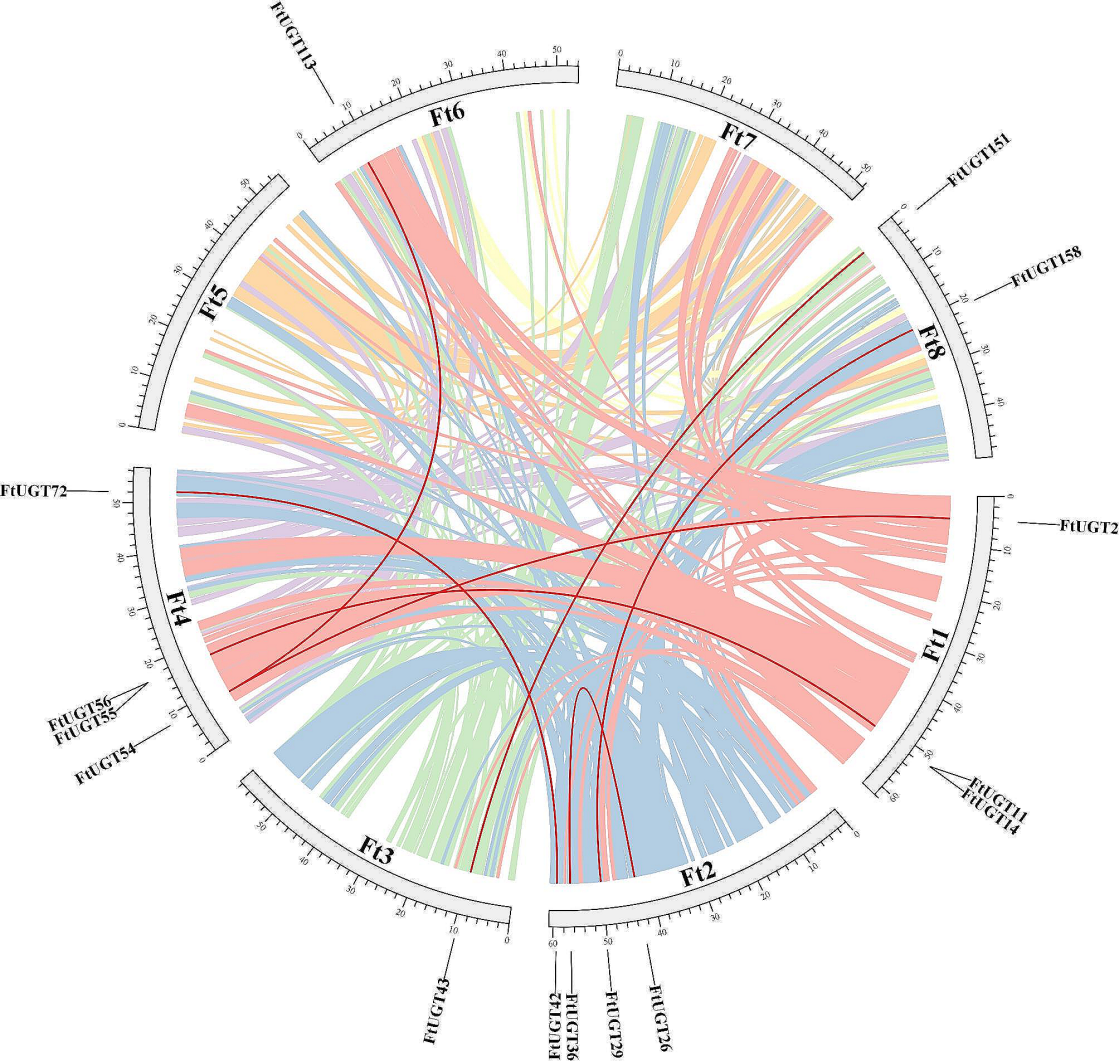
Chromosome localization of duplicated *FtUGTs* in *F. tataricum*. The red lines represent the segmentally duplicated genes and the colored bands represent the collinear block in the genome of *F. tataricum*

Furthermore, there were eight pairs of segmental duplications, which were found to be heterogeneously distributed across the eight chromosomes. Similar to the tandem duplications, segmental duplication pairs were also typically members of the same UGT family (Figs. 3 and 4, Additional file 5: Table S5). It was worth noting that *FtUGT54* was involved in two distinct segmental duplication events (*FtUGT54* with *FtUGT2/FtUGT113*). Additionally, several *FtUGTs* were determined to be products of both tandem and segmental duplication events. In general, gene duplication may have served as a primary driving force in the evolution of *FtUGTs*.

### Evolution collinearity of FtUGTs between F. Tataricum and other plant species

**Fig. 5:**
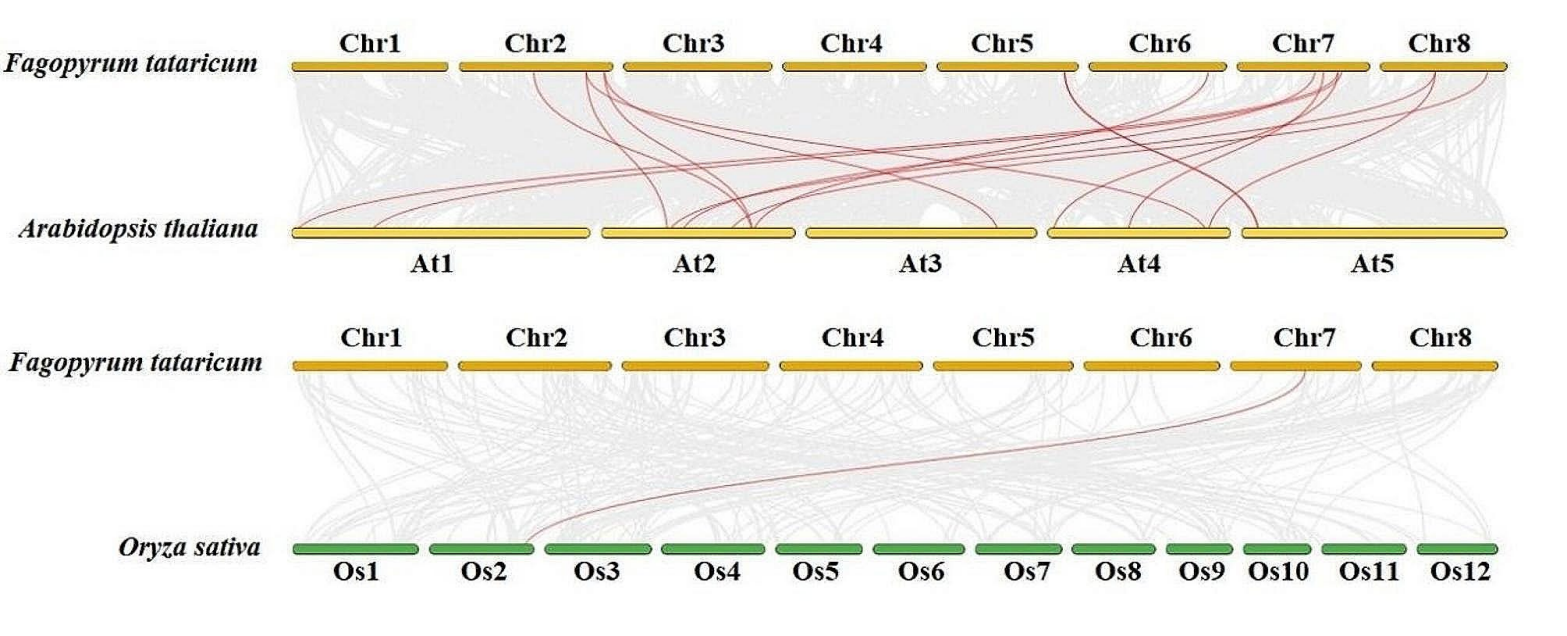
Collinearity analyses of *UGTs* between *F. tataricum* and two representative plant species (*Arabidopsis thaliana*, *Oryza sativa* subsp. *indica*). Gray lines indicate the collinear blocks within *F. tataricum* and other plant species and red lines highlight homologous *UGT* gene pairs

To investigate the evolutionary relationship of *FtUGTs* with other plant species, we conducted a thorough analysis of collinearity involving Arabidopsis and rice, which are widely recognized as monocotyledonous and dicotyledonous model plants, respectively (Fig. 5, Additional file 6: Table S6). As anticipated, a greater number of homologous genes (16 *UGTs*) were observed between *F. tataricum* and *A. thaliana*, whereas only one pair of homologous genes was found to exist between *F. tataricum* and *O*. *sativa*. It was also observed that some of the homologous genes were situated within the expansion blocks of the chromosomes. For instance, *FtUGT32, FtUGT99*, *FtUGT102*, *FtUGT134*, *FtUGT136*, and *FtUGT159* were all found to belong to the tandem repeat blocks. To acquire a better comprehension of the evolutionary constraints governing the *FtUGT* superfamily, Tajima’s D-neutral test was applied to the *FtUGTs* [54, 55]. The outcome of the test yielded a D = 2.19, with the deviation from zero suggesting that the *FtUGT* superfamily may have undergone selective pressure for purification during the course of its evolution. (Additional file 7: Table S7).

### Evolutionary analysis of UGTs among different plant species

**Fig. 6:**
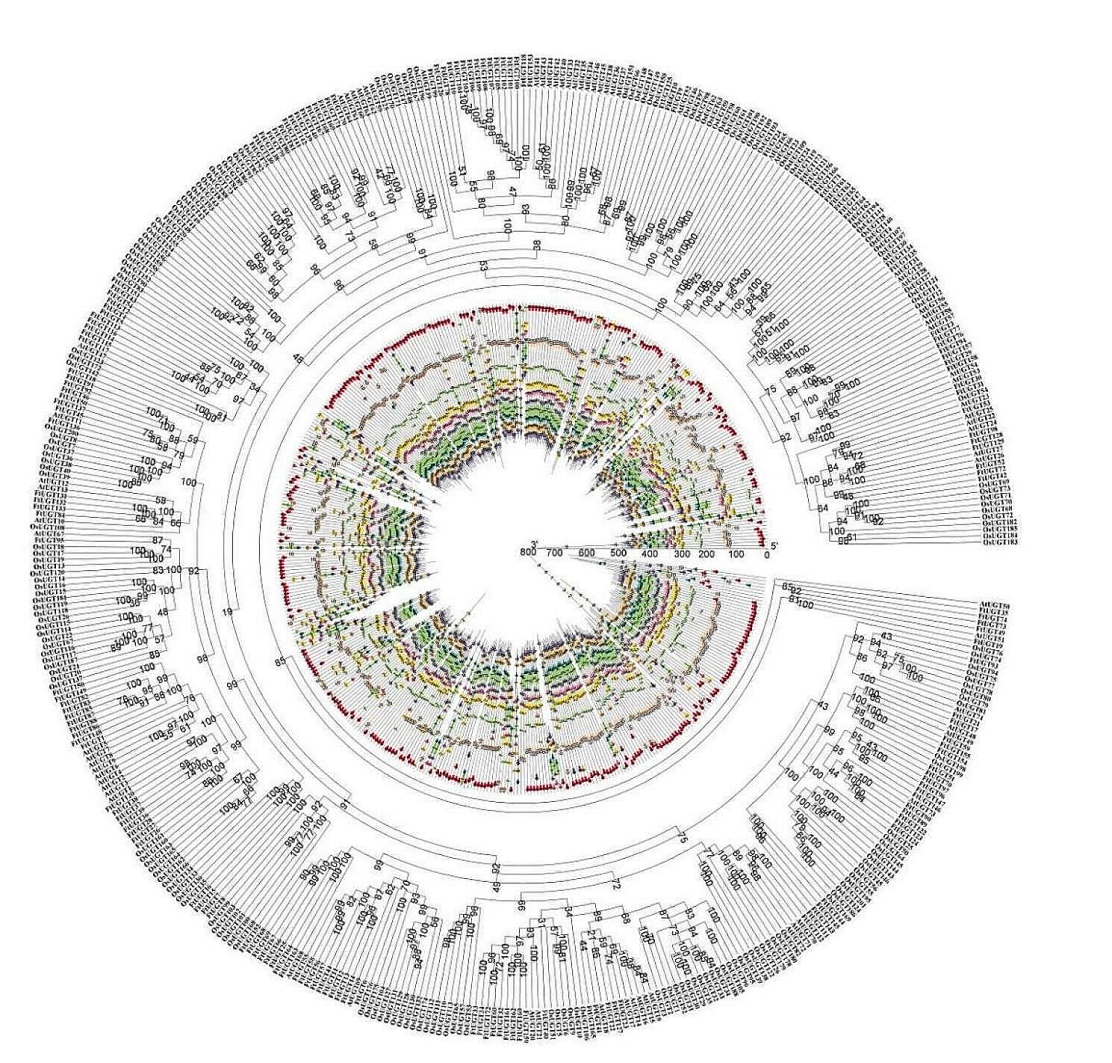
Phylogenetic relationships and motifs composition of UGTs in *F. tataricum*, *A. thaliana*, and *O. sativa*. Outer panel: Unrooted phylogenetic tree constructed using Geneious R11. Inner panel: Distribution of the conserved motifs in UGTs. The different color boxes represent different motifs

The evolutionary relationships among UGTs in *F. tataricum*, *A. thaliana*, and *O. sativa* were analyzed by constructing an unrooted tree based on a total of identified 173 FtUGTs, 73 AtUGTs, and 201 OsUGTs, which was generated using the NJ method of Geneious R11 (Fig. 6, Additional file 2: Table S2). Through the use of the MEME web server, a total of ten conserved motifs were identified in the UGTs of *F. tataricum*, *A. thaliana*, and *O. sativa*. The majority of UGTs were found to harbor motifs 1, 3, and 4, further highlighting their functional significance across different plant species. From the perspective of motif structure across different UGT families, it was observed that the families UGT71, UGT78, and UGT86 exhibited greater diversity, while the families UGT76, UGT79, UGT85, and UGT95 showed greater conservation across various plant species suggesting a possible functional similarity amongst the UGTs. Based on the distribution of motifs across the UGT proteins, it was observed that certain motifs were found only at specific positions. For instance, Motif 7 was located between motifs 10 and 2, while Motif 5 was consistently positioned at the N-terminus of the protein. In contrast, Motifs 8 and 9 were predominantly located at the C-terminus of the protein.

### Expression analysis of FtUGTs in various tissues

**Fig. 7:**
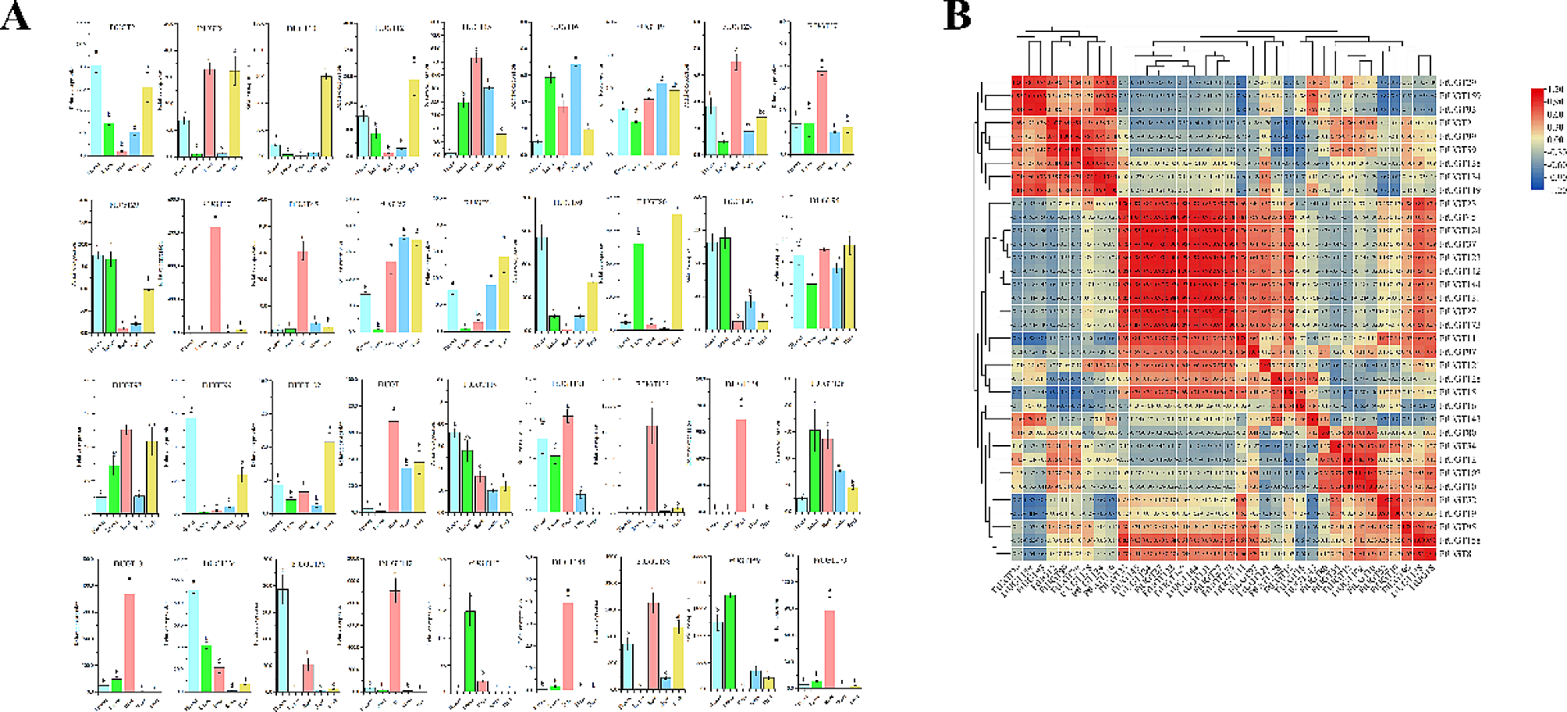
Expression analysis of *FtUGTs* in different tissues and the correlation between their expression patterns. **A**. Expression patterns of 36 *FtUGTs* in flowers, leaves, roots, stems, and fruits were examined by qRT-PCR. Different letters above columns indicate statistically significant differences between tissues (LSD test, *P* < 0.05). Error bars represent SE (*n* = 3). **B**. Positive number: positively correlated; negative number: negatively correlated

To gain insight into the potential biological functions of *FtUGTs*, an investigation was conducted into the expression patterns of 36 *FtUGTs* belonging to different families. To ensure a representative sample, we randomly selected genes from each family, making sure that every family is represented by at least one gene. For families with more members, we selected 2–3 genes to provide a more comprehensive analysis. The expression levels of these genes in various organs of *F. tataricum*, including roots, stems, leaves, flowers and fruits, were determined through qRT-PCR analysis (Fig. 7A). The data revealed that the expression patterns of the 36 *FtUGTs* varied across different organs of *F. tataricum*. 19 genes displayed the highest expression levels in roots, while nine genes exhibited the highest expression in fruits. Additionally, eight genes were found to be highly expressed in flowers, six genes showed highest expression levels in leaves, and four genes were highly expressed in stems. The diverse expression patterns observed amongst *FtUGTs* suggest that they are likely to participate in multiple roles throughout plant development. Correlations between expression patterns of *FtUGTs* were evaluated using a heat map, which revealed that significant correlations were established between the expression patterns of certain *FtUGTs*, implying that they might exert potential synergistic effects (Fig. 7B). Most of the *FtUGTs* tested displayed a significant positive correlation. For instance, genes including *FtUGT29*, *FtUGT93*, and *FtUGT159* exhibited high expression levels in both flowers and leaves. Additionally, genes such as *FtUGT10*, *FtUGT12*, *FtUGT54*, and *FtUGT102* exhibited high expression in fruits. Moreover, the expression patterns of genes like *FtUGT23*, *FtUGT27*, *FtUGT37*, *FtUGT45*, *FtUGT111*, *FtUGT123*, *FtUGT124*, *FtUGT131*, *FtUGT142*, *FtUGT144*, and *FtUGT173* were highly expressed in roots.

### Expression analysis of FtUGTs at different developmental stages of fruits in F. Tataricum

**Fig. 8:**
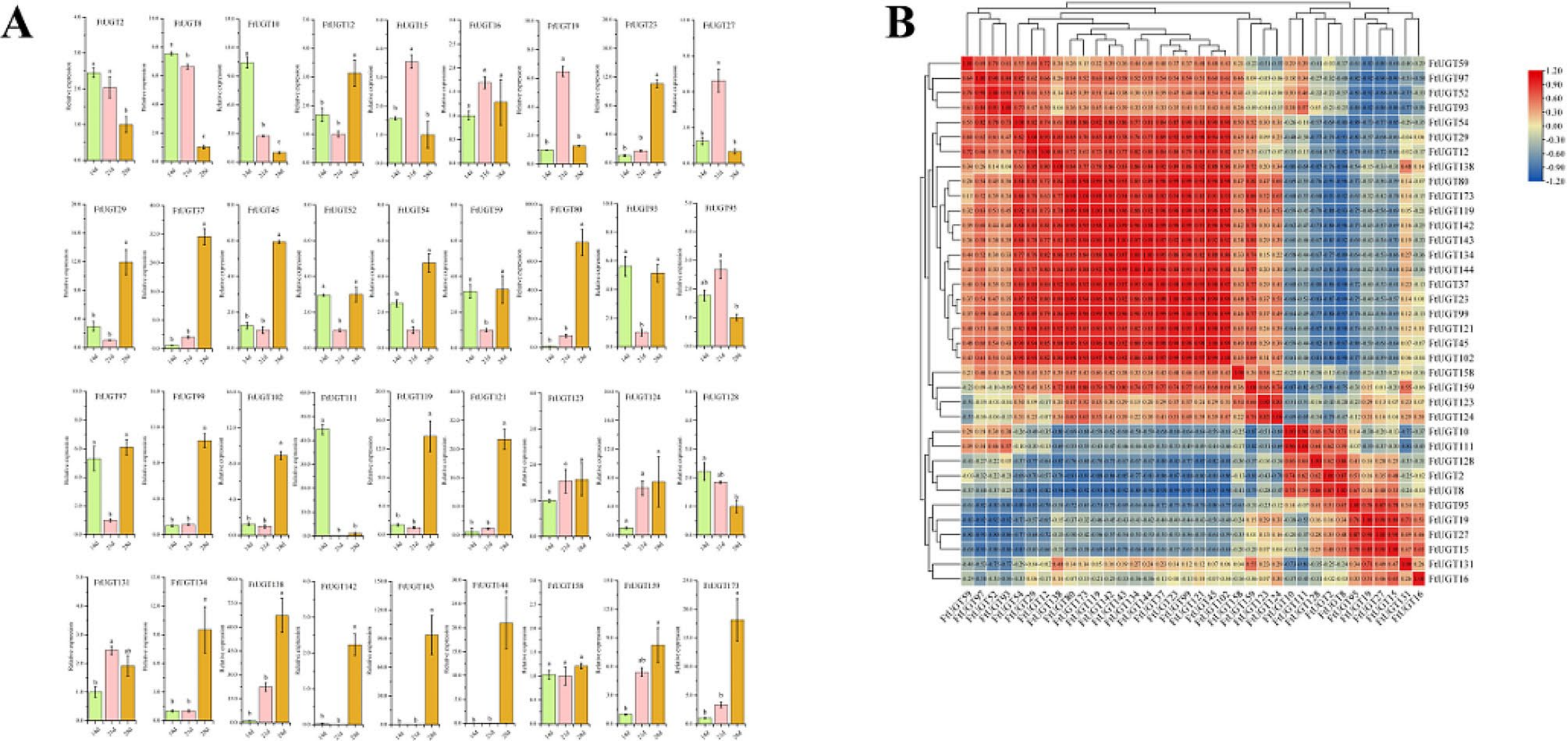
Expression analysis of *FtUGTs* at different developmental stages of fruits and the correlation between their expression patterns. **A**. Expression patterns of *FtUGTs* at different developmental stages of fruits (i.e., 14, 21, and 28 days after pollination). Different letters above columns indicate statistically significant differences between tissues (LSD test, *P* < 0.05). Error bars represent SE (*n* = 3). **B**. Positive number: positively correlated; negative number: negatively correlated

**Fig. 9:**
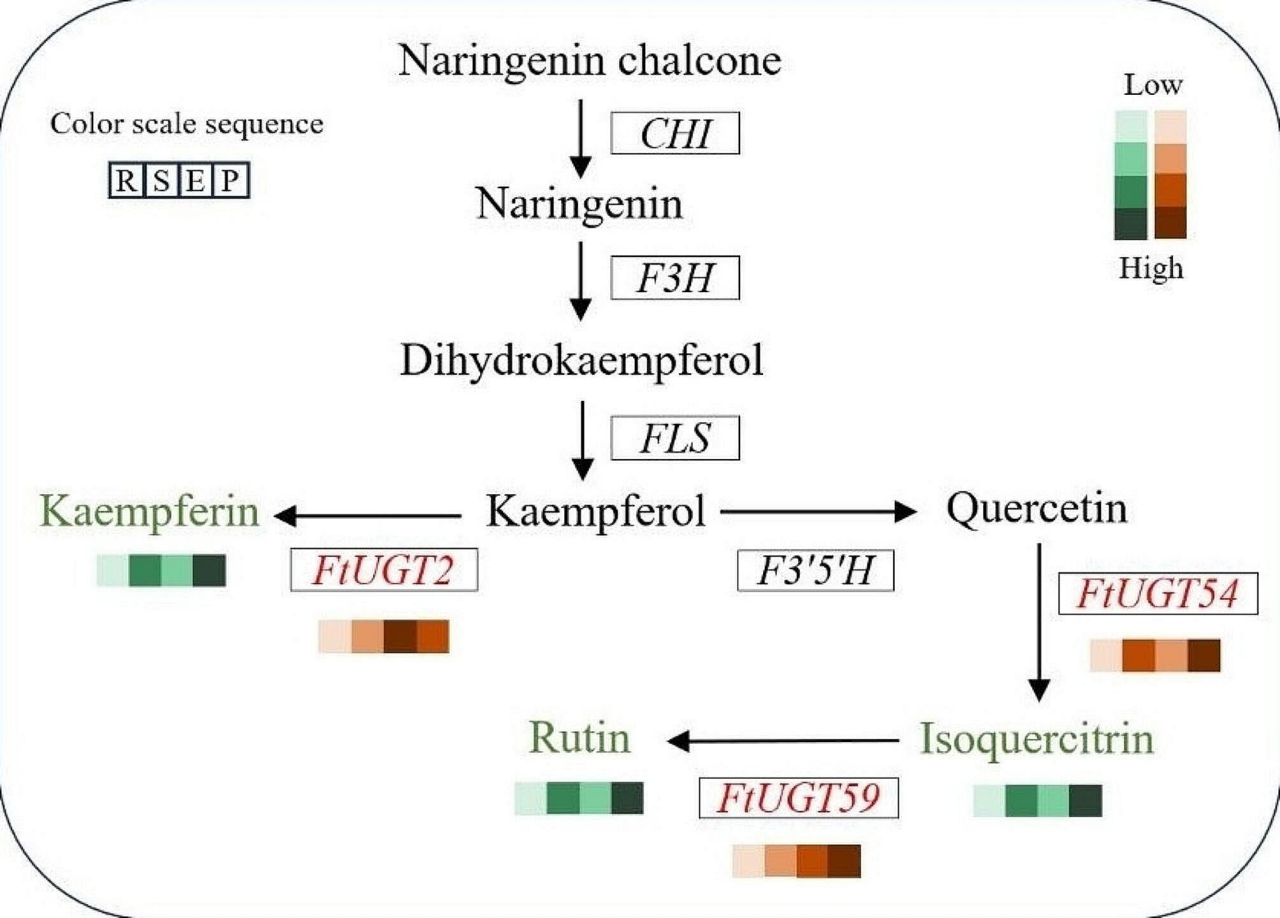
Combined analysis of the expression level of *FtUGTs* and the content of flavonoids in different tissues at different developmental stages. The expression level of genes and the content of metabolites are represented using the color scale. The color scale sequence is as follows: R, roots; S, shoots; E, fruits at the early-filling stage; P, fruits at the post-filling stage

In order to understand the roles of *FtUGTs* in fruits development of *F. tataricum*, we further performed qRT-PCR to evaluate the expression levels of *FtUGTs* at different developmental stages of fruits (i.e., 14, 21, and 28 days after pollination) of *F. tataricum.* The results showed that the expression patterns of *FtUGT* genes were inconsistent in fruits at 14, 21, and 28 days (Fig. 8A). *FtUGT2*, *FtUGT8*, *FtUGT10*, *FtUGT111*, and *FtUGT128* were highly expressed at the initial filling stage (14 d). *FtUGT15*, *FtUGT19*, and *FtUGT27* expression reached the highest levels at the middle-filling stage (21 d), and most *FtUGTs* (18 genes) were highly expressed at the post-filling stage (28 d). It is speculated that the glycosylation of a wide range of bioactive compounds (such as flavonoids and polyphenols) may be carried out at the post-filling stage. To confirm these findings, we performed a combined analysis of *FtUGTs* gene expression and the biosynthetic pathways of secondary metabolites (such as flavonoids) present in the fruits. Recent studies have shown that the fruits of *F. tataricum* contain flavonoids such as rutin, isoquercitrin, quercetin, kaempferin, dihydrokaempferol, naringenin, and naringenin chalcone [51]. we mapped out the biosynthetic pathways of these metabolites through the use of the KEGG online software and discovered that UGTs is involved in the glycosylation reactions during the synthesis of rutin, isoquercitrin, and kaempferin (Fig. 9). Through homology analysis, we identified potential *UGTs* (*FtUGT2*, *FtUGT54*, and *FtUGT59*) in *F. tataricum*. We analyzed the expression patterns of these genes in roots, stems, fruits at the early-filling stage, and fruits at the post-filling stage. It is found that *FtUGT2*, *FtUGT54*, and *FtUGT59* were highly expressed in fruits at the post-filling stage and the content of downstream metabolites kaempferin, isoquercitrin, and rutin were high in fruits at the post-filling stage (Fig. 9). In other words, the gene expression levels were closely linked with the content of metabolites found in the fruits. To determine the potential regulatory relationship among *FtUGTs* in different fruit-filling stages, a heat map of their expression patterns was constructed based on the level of expression in different fruit development processes (Fig. 8B). A total of 17 *FtUGTs* (*FtUGT12*, *FtUGT23*, *FtUGT29*, *FtUGT37*, *FtUGT45*, *FtUGT54*, *FtUGT80*, *FtUGT99*, *FtUGT102*, *FtUGT119*, *FtUGT121*, *FtUGT134*, *FtUGT138*, *FtUGT142*, *FtUGT143*, *FtUGT144*, and *FtUGT173*) were identified to exhibit significant positive correlation matrix, indicating that their expression may act synergistically and contribute to the formation of a complex regulatory network during the filling process of *F. tataricum*.

### Expression analysis of FtUGTs in response to drought and cadmium stress in F. Tataricum

**Fig. 10:**
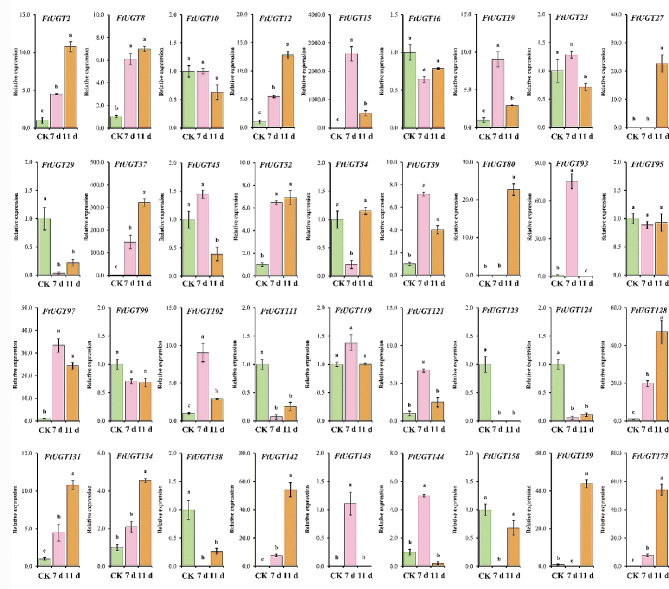
Expression analysis of *FtUGTs* under drought at the seedling stage. Different letters above columns indicate statistically significant differences between tissues (LSD test, *P* < 0.05). Error bars represent SE (*n* = 3)

**Fig. 11:**
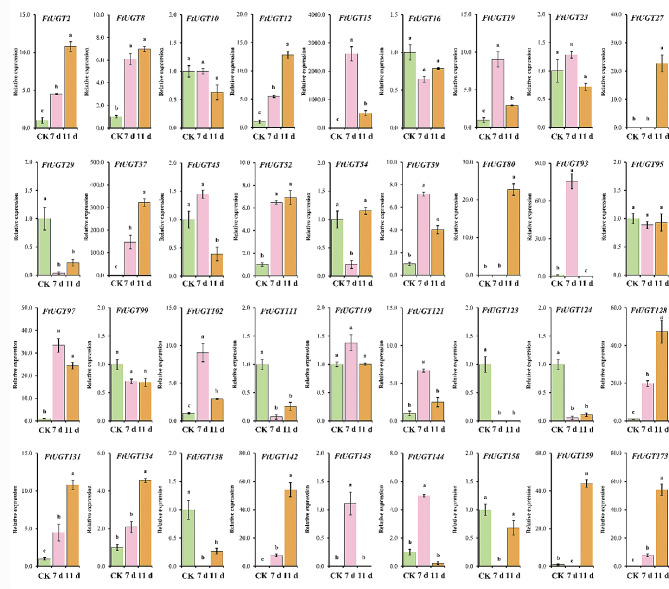
Expression analysis of *FtUGTs* under cadmium stress at the seedling stage. Different letters above columns indicate statistically significant differences between tissues (LSD test, *P* < 0.05). Error bars represent SE (*n* = 3)

To further explore the involvement of *FtUGTs* in the response of *F. tataricum* to drought and cadmium stress, we conducted qRT-PCR assays to examine the expression levels of these genes under PEG stress and CdCl_2_ stress (Figs. 10 and 11). Our findings revealed that certain *FtUGTs* were significantly modulated in response to different stress conditions, displaying either upregulation or downregulation in expression levels.

In response to drought stress, a total of 14 *FtUGTs* were observed to be significantly upregulated at both 7 days and 11 days of the stress condition. These genes included *FtUGT2*, *FtUGT8*, *FtUGT12*, *FtUGT15*, *FtUGT19*, *FtUGT102*, *FtUGT121*, *FtUGT128*, *FtUGT131*, *FtUGT142*, *FtUGT143*, *FtUGT144*, *FtUGT159*, and *FtUGT173*. Of the remaining genes, three *FtUGTs* (*FtUGT29*, *FtUGT138*, and *FtUGT158*) were significantly downregulated at both 7 days and 11 days. 15 *FtUGTs* demonstrated altered expression levels over time. In contrast, only three FtUGTs exhibited no change in expression levels under drought stress.

Upon exposure to cadmium stress, 12 *FtUGTs* were found to be significantly upregulated at both 7 days and 11 days. Conversely, five genes were notably downregulated at both time points. Furthermore, the expression levels of 11 *FtUGTs* were observed to fluctuate over time, whereas the expression of eight genes remained unchanged. Collectively, these observations suggest that certain *FtUGTs* play a critical role in the response of *F. tataricum* to both drought and cadmium stress conditions.

## Discussion

Within plants, glycosylation is recognized as an essential modification of secondary metabolites that is critical to numerous cellular processes and maintaining proper cellular homeostasis. This process is mediated by GTs, which can be classified into 115 different families. The largest of these GT families is family 1, which is commonly referred to as UGTs. UGTs represent a large multigene superfamily that plays a key role in glycosylation across the plant kingdom. Functional studies of UGTs within plants have revealed their involvement in regulating growth and development, as well as their response to environmental stresses. Due to advancements in genome sequencing, the UGT gene superfamily has been identified in numerous plant species [10, 18–26]. Despite its growing popularity as a minor crop due to its potential health benefits, *F. tataricum* has yet to be thoroughly examined in regards to its *UGT* gene superfamily.

To better understand the *UGT* gene superfamily within *F. tataricum*, we searched the predicted peptide databases using the conserved UDPGT domain and the PSPG motif consisting of 44 amino acids as our query. This comprehensive analysis allowed us to examine phylogenetic relationships, conserved motifs, gene structure, cis-acting elements, gene duplication. Our findings revealed that the *FtUGT* gene superfamily is considerably large and diverse.

### UGT is a large multigene family

A total of 173 *FtUGT* genes were identified. The number of *FtUGTs* in *F. tataricum* was higher than that of *Zea mays* (147) [22], *Brassica rapa* (147) [52], *A. thaliana* (107) [10], and *Cicer arietinum* (96) [53]. However, it was lower than that of *Oryza sativa* (200) [54], *Gossypium hirsutum* (196) [23], and *Triticum aestivum* (179) [55]. The ratio of *FtUGTs* to the total genes of *F. tataricum* was 0.47%, which exceeded that of *Z. mays* (0.46%) [22], *(A) thaliana* (0.39%) [10], *G. hirsutum* (0.38%) [23], and *(B) rapa* (0.33%) [52], but lower than that in *O*. *sativa* (0.53%) [54].

### Phylogeny: structure–function relatedness of FtUGTs

All FtUGTs showed significant structural differences, indicating that gene functions are highly diverse. The length of the FtUGTs varied from 123 to 645 amino acids and the majority of FtUGT proteins had a size range of 400 to 500 amino acids, which account for the length stability of FtUGTs. The majority of FtUGTs had a pI range of 5 to 6, indicating that pI of FtUGTs is relatively stable.

Through our phylogenetic analysis, we were able to identify 21 distinct families within *FtUGT* gene superfamily. Notably, at least one FtUGT belonging to each *A. thaliana* family was identified, indicating that these UGT families have been preserved throughout the course of long-term evolution and likely play fundamental biological roles. Furthermore, our findings provide support for the hypothesis that the separation of UGT families may have occurred before the differentiation between *F. tataricum* and *A. thaliana*. Interestingly, some new family (UGT93, UGT94, and UGT95) and genes (*FtUGT35*, *FtUGT73*, and *FtUGT74*) have been generated with evolution. Among them, UGT93 may come from the separation of UGT71, UGT72, and UGT88. *F. tataricum* have abundant secondary metabolites [56], such as flavonoid glycosides (rutin, nicotilorin, kaempferol-3-O-glucoside, and quercetin-3-O-glucoside). The new families may be related to the formation of these secondary metabolites, requiring further validation experiments.

Among the 21 families, UGT71 had the highest number of members (18, 10.4%) also with the most tandem duplication events. UGT88 had the fewest members. As observed in other plant species [10, 18–26], the diverse UGT families within *F. tataricum* may possess distinct differentiation abilities throughout the course of long-term evolutionary processes. Furthermore, variations within these genes are likely to play a pivotal role in their diverse functions across different species.

Our examination of the conserved amino acid sequence of FtUGTs has identified ten distinct conserved motifs, among which Motif 1 was the PSPG motif ensuring the conservation of the C-terminal which is is consistent with the function of recognizing similar or identical UDP-sugar donors in most FtUGTs. Notably, the N-terminal region of FtUGTs is highly diverse, resulting from a rich variety of receptor. Furthermore, we observed that several FtUGTs undergo motif loss events, indicating potential structural modifications over time that could result in functional differences among these genes.

Our analysis revealed that a majority of *FtUGTs* (142, or 82.1%) either lacked introns or contained only one intron (Fig. 2A). Notably, the proportion of *FtUGTs* without introns (70, 40.5%) was found to be similar to that observed in other plant species, such as *Linum usitatissimum* (40.1%) [20], Petunia (44.2%) [57], and cassava (47.1%) [58]. Given that genes lacking introns do not require extensive post-transcriptional and post-translational modification [59, 60], they can rapidly and continuously produce functional proteins, resulting in their quick response to stress. This could help explain why UGTs are highly active during plant growth, development, and in response to environmental stress.

The process of gene amplification has played a pivotal role as the evolutionary engine of plant genomes, contributing to adaptability to different environmental conditions and differentiation among various species [61]. Within our study, we observed 39 tandem duplication events, involving 70 *FtUGTs* (approximately 40.5%). Notably, the majority of these events occurred specifically within certain families (UGT71, UGT85, UGT73, UGT76, and UGT89), indicating that these families exhibit a strong bias toward tandem duplication events. Of particular interest is the identification of a narrow region on Chr5, spanning just 60.9 kb, which contained four pairs of tandem duplications (*FtUGT99*, *FtUGT100*, *FtUGT101*, *FtUGT102*, *FtUGT103*, *FtUGT104*, *FtUGT106*, and *FtUGT107*), all of which belonged to UGT76 family. This observation suggests that chromosome recombination within this region is highly active, fueling the duplication of genes within this region and contributing to the expansion of UGT76 family genes within *F. tataricum* (Fig. 3). All genes with tandem repeats belonged to the same family, suggesting a lack of gene exchange between different UGT families. Furthermore, we found that tandem duplications played a more significant role in the amplification of *FtUGTs*, accounting for 70 genes, as compared to segmental duplications, which were responsible for only 15 genes. Notably, segmental duplication events were also found to have a preference for specific families (UGT78 and UGT89).

### Expression patterns and functional prediction of FtUGTs

One approach to predicting the physiological functions of genes involves analyzing gene expression patterns, which can provide valuable insights into their roles in plant growth and development. Consistent with previous research showing the widespread involvement of *UGTs* in these processes, our findings suggest that the expression of *FtUGTs* is often correlated with specific physiological functions. For instance, the expression of *FtUGT59* was found to be particularly high in flowers, a pattern that is similar to its homologous gene, *UGT79B31*, in *Petunia hybrida*. *UGT79B31* has been identified as being responsible for the terminal modification of pollen-specific flavonols, a crucial process for regulating pollen fertility [62]. *FtUGT52*, a member of the UGT74 family, was found to be highly expressed in roots, stems, and fruits, a pattern that is similar to *OsUGT74J1* in rice. *OsUGT74J1* controls salicylic acid homeostasis to alter resistance to rice blast [63]. Similarly, we found that *FtUGT159* was highly expressed in flowers and leaves, consistent with the expression pattern of its homologous gene *AT2G15480*. Research suggests that *AT2G15480* may play crucial roles in response to drought and high-temperature stresses [64]. It is observed that both *FtUGT23* and its homologous gene *AT2G36750* are expressed at high levels in roots, with research indicating that *AT2G36750* in particular can regulate the growth of roots in *Arabidopsis* through auxin signal transduction processes in response to environmental signals [65]. Similarly, the expression of *FtUGT102* was found to be elevated in fruits, a pattern similar to that observed in its homologous gene, *AT5G05900*. Both *FtUGT102* and *AT5G05900* belong to the UGT76 family, with *AT5G05900* being specifically expressed in siliques during the filling stage and responded to heat stress in *Arabidopsis* [66]. These findings can serve as a valuable starting point to further explore the functions of these genes and their potential roles in stress response and growth regulation within *F. tataricum* and other plant species.

As *F. tataricum* fruit is a source of various bioactive compounds with established health benefits, our study evaluated the expression levels of *FtUGTs* at different fruit developmental stages and associated gene expression pattern with biosynthetic pathways of flavonoids present in fruits (Figs. 8 and 9). This provides insights into the molecular mechanisms underlying the production of these compounds within fruits. By identifying specific *FtUGTs* that are expressed at high levels during specific developmental stages, our findings may facilitate the development of new *F. tataricum* varieties that contain high levels of beneficial bioactive compounds in their fruits. This could have a significant impact on the functional food industry and contribute to improved human health.

To better understand the role of *FtUGTs* in aiding *F. tataricum*’s adaptation to different environmental conditions, we conducted a comprehensive analysis of the expression patterns of 36 *FtUGTs* during seedling stage under various stressors (Figs. 10 and 11). Notably, our findings revealed that the expression of 33 FtUGTs was significantly regulated in response to drought stress, consistent with *F. tataricum*’s reputation as a drought-tolerant crop. *FtUGT128*, a member of the UGT74 family, was significantly upregulated in response to drought stress at both 7 and 11 days. Similarly, a homologous gene to *FtUGT128*, *UGT74E2* (*AT2G23250*), has been found to regulate Arabidopsis architecture and water stress tolerance by perturbating indole-3-butyric acid (IBA). Transgenic UGT74E2OE plants have been shown to exhibit increased tolerance to both salinity and drought stress compared with wild-type plants [67]. We also observed that *FtUGT159* was significantly upregulated in response to drought stress at both 7 and 11 days, consistent with the response pattern of its homologous gene *AT2G15480*. Research has suggested that *AT2G15480* plays a crucial role in response to drought stress [64, 68]. In addition to their response to drought stress, we also found that 28 out of 36 *FtUGTs* were regulated in response to cadmium stress. These observations suggest that *FtUGTs* may play a crucial role in helping *F. tataricum* adapt to cadmium stress, a problem that stems from heavy metal pollution and can have detrimental effects on the growth and survival of plants. Our study thus provides valuable insights into the physiological role of *FtUGTs* in environmental adaptation, which could help inform future research into developing crop varieties that are more resistant to various environmental stressors.

## Conclusions

Our study of the *UGT* superfamily in *F. tataricum* revealed the presence of 173 members, all of which possess the characteristic PSPG domain region. In addition to characterizing their physical features, we conducted a comprehensive analysis of *FtUGTs* evolutionary relationships, gene structure, conserved motifs, and gene replication events. Our findings demonstrated that *FtUGTs* within the same family typically share a similar gene structure and motif composition, with the majority of these genes lacking introns or containing no more than one intron. We also found that tandem repeats were more important than segmental duplications in the amplification of *FtUGTs*.

To better understand the functional characteristics of members of the *UGT* superfamily within *F. tataricum*, we employed quantitative reverse transcription PCR to analyze the expression patterns of 36 representative *FtUGTs* across different tissues and fruit development stages, as well as their response to two abiotic stressors. Our study revealed that *FtUGTs* are widely involved in various physiological processes including growth, grain development, and response to environmental stresses. As such, Our results provide a theoretical basis for further exploration of the functional characteristics of members of *UGT* superfamily and for understanding the growth, development, and metabolic model of this important crop species.

## Materials and methods

### Plant materials and growth conditions

The *F. tataricum* variety “Pinku1” materials used in the experiment were supplied by Prof. Dongao Huo of Taiyuan Normal University. “Pinku1” has been grown in the greenhouse at Taiyuan Normal University since 2022. *F. tataricum* plants were grown in pots filled with soil and vermiculite (1:1) in a growth room with a 12 h/26℃ day and 12 h/22℃ night regime, and relative humidity of 80%. We collected the roots, stems, leaves, flowers, and grains separately from five plants with good growth and similar growth conditions, and quickly placed them in liquid nitrogen for storage at -80 °C for further use. Grain samples were collected at 14D, 21D, and 28D respectively.

*F. tataricum* seedlings with 7–8 leaves were treated in the 400 mg/L PEG solution and 10 mg/L CdCl_2_ solution, respectively. After a 7-day or 11-day treatment, leaves were taken up for RNA extraction.

### Total RNA extraction, cDNA reverse transcription, and qRT-PCR analysis

To generate cDNA for our analyses, we utilized RNAprep Pure Polysaccharide Polyphenol Plant Total RNA Extraction Kit (DP441) and FastKing one-step reverse transcription fluorescence quantitative kit (probe method) (FP314). The gene expression of *FtUGTs* was analyzed by qRT-PCR, and the primer designed by Primer 5.0 (Additional File 8: Table S8). The *FtH3* gene is selected as an internal control, which is stably expressed in almost all tissues at each growth stage [69]. Correlation of expressed data according to 2^−(ΔΔCT)^ method.

### Gene identification

We downloaded the *F. tataricum* whole genome sequence information from the Molecular Breeding Knowledgebase (MBKbase) (http://mbkbase.org/Pinku1/). Concerning the UGT protein sequence of *Arabidopsis*, the BLASTp program was used to identify candidate target proteins (score value ≥ 100 and e-value ≤ 1e − 10). Then, the Hidden Markov Model (HMM) files corresponding to the UDPGT domain (PF00201) and PSPG motif from the PFAM protein family database(http://pfam.xfam.org/) was downloaded [70]. We have used two HMMER3.0 (default parameter) with a cutoff of 0.01 (http://plants.ensembl.org/hmmer/index.html) [70], and SMART (http://smart.embl-heidelberg.de/) to determine the existence of the UDPGT domain and PSPG motif [71, 72]. The sequence length, molecular weight, pI, and subcellular localization of 173 FtUGT proteins were obtained using the tools on the ExPASy website (https://web.expasy.org/compute_pi/).

### UGT gene structures

To understand the structural differences between FtUGTs, the conserved motifs in 173 FtUGT proteins were further observed [73]. Based on CDS length and corresponding full-length sequence, a gene structure display server (GSDS: http://gsds.cbi.pku.edu.cn) was used to analyze the exon-intron structure of all *FtUGTs.* An online MEME program (http:/meme.nbcr.net/meme/intro.html) was used to analyze protein sequences, with the following parameters: the optimal motif width is 6 ~ 200, and the maximum motif number is 10.

### Chromosomal distribution and gene duplication

Based on the physical location information of the genome database, all *FtUGT* genes are mapped to the *F. tataricum* genome. Multiple collinear scanning toolkits (MCScanX) were used to analyze *FtUGT* gene’s segmental replication events based on default parameters [74]. We used the Dual Synteny plotter (https://github.com/CJ-Chen/TBtools) to analyze the *UGT* gene homology between *F. tataricum* and two model plants (*A. thaliana* and *O. sativa* subsp. *indica*).

### Phylogenetic analysis and classification of the FtUGT gene family

These FtUGT proteins were grouped according to the classification of the AtUGTs [75–78]. The phylogenetic trees were built using the NJ method of MEGA 11.0 via Geneious R11, with the following parameters: Jukes-Cantor model, global alignment with free end gaps, and a bootstrap value of 1000. The full-length amino acid sequences of the UGT proteins derived from *A. thaliana*, *O. sativa* subsp. *indica*, combined with the newly identified FtUGTs were used for phylogenetic analysis.

### Electronic supplementary material

Below is the link to the electronic supplementary material.

Supplementary Material 1

Supplementary Material 2

Supplementary Material 3

Supplementary Material 4

Supplementary Material 5

Supplementary Material 6

Supplementary Material 7

Supplementary Material 8

## Data Availability

The complete information on Tartary buckwheat genome sequence (Pinku1 Genome sequence) and the datasets analysed during the current study are available in the Molecular Breeding Knowledgebase (MBKbase) (http://www.mbkbase.org/Pinku1/). All data analyzed during this study are included in the article and its supplementary information files.

## Abbreviations

At: *Arabidopsis thaliana*
Os: *Oryza sativa*
AtUGT: *Arabidopsis thaliana* UGT
OsUGT: *Oryza sativa* UGT
FtUGT: *Fagopyrum tataricum* UGT
qRT-PCR: Quantitative real-time polymerase chain reaction
TF: Transcription factor
CDS: Coding sequence
HMM: Hidden Markov Model
pI: Isoelectric point
BLAST: Basic local alignment search tool
